# Supplementary material for: Efficient Base-Catalyzed Kemp Elimination in an Engineered Ancestral Enzyme
Source: Int J Mol Sci. 2022 Aug 11;23(16):8934. doi: 10.3390/ijms23168934 (PMC9408544; doi:10.3390/ijms23168934)
Supplement: Supplementary file 1 [file ijms-23-08934-s001.zip › ijms-1863460-supplementary.pdf]

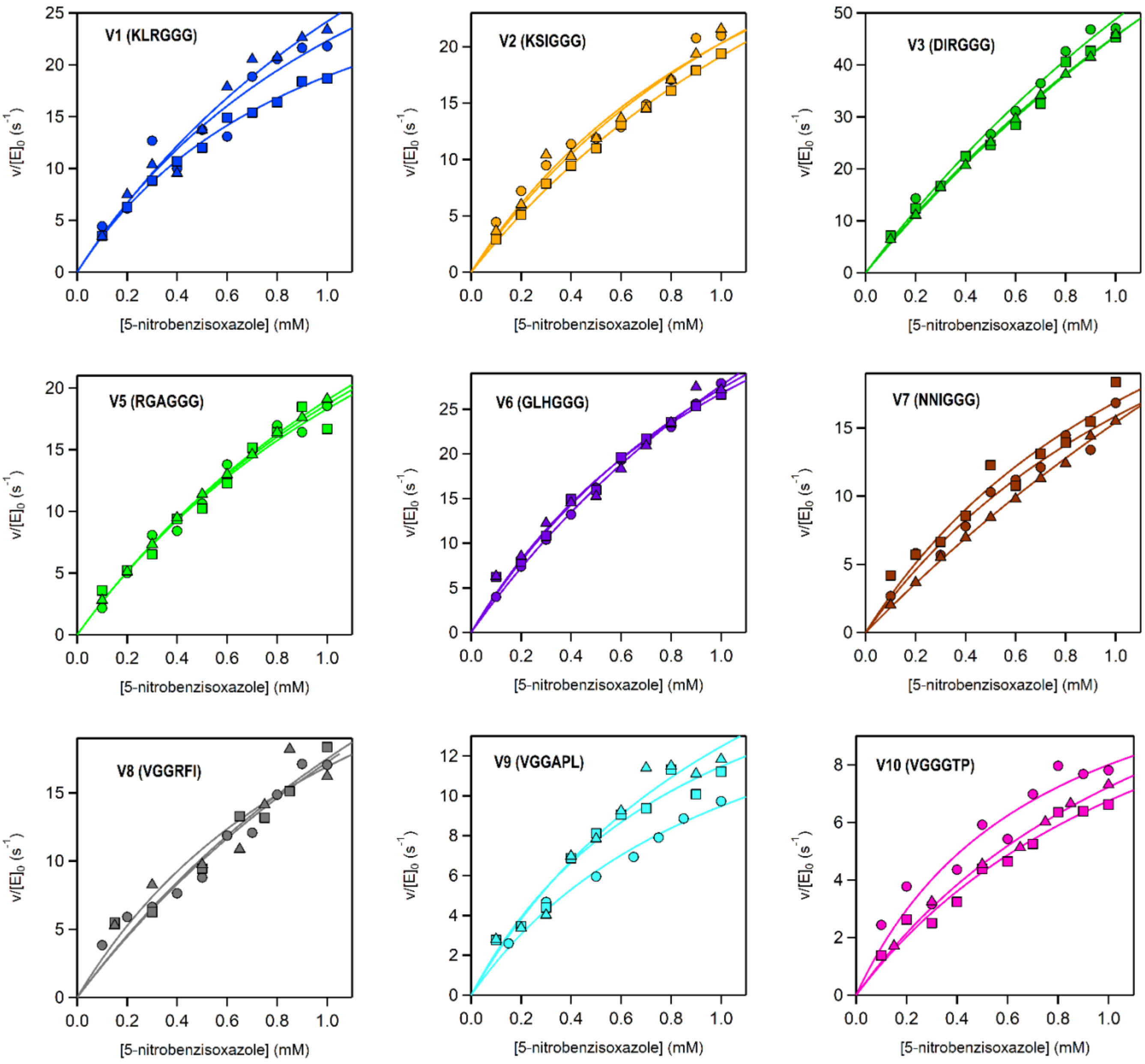

Figure S1. Michaelis-Menten profiles of the three independent determinations for the top variants from the primary library screening at pH 7. Note that the Michaelis-Menten profiles of the best variant (V4) are not included here (see Figure 2 in the main text). The sequences at the relevant section of the included polypeptide are shown for each variant.

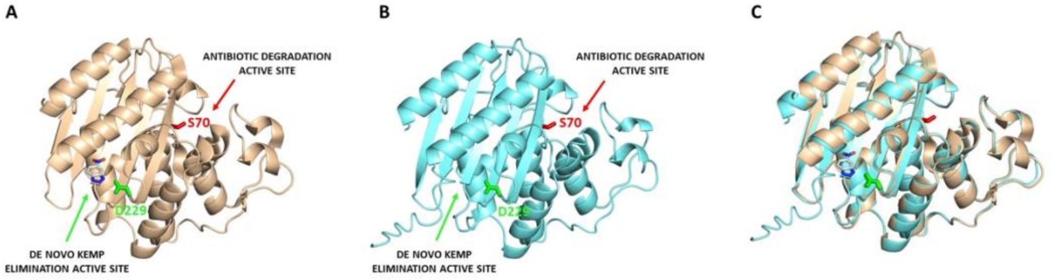

Figure S2. (A) Structure (PDB ID 5FQK) of the W229D/F290W variant of an ancestral β-lactamase with Kemp eliminase activity [33] showing location of the *de novo* Kemp elimination active site (identified by the bound transition-state analogue and the catalytic base D229) and the antibiotic (β-lactam) degradation active site (identified by the catalytic S70).**(B)** Same as in (A) but for the structure predicted by AlphaFold2 for the best Kemp eliminase found in this work. **(C)** Superposition of the experimental and predicted structures shown in (A) and (B).

Table S1. Catalytic parameters for the cleavage of 5-nitrobenzisoxazole at pH 7 (HEPES 10 mM NaCl 100 mM) and 1% acetonitrile and 25 ºC catalyzed by the engineered and evolved versions of Precambrian β-lactamases. Values of catalytic parameters derived from the fitting of the Michaelis-Menten equation are given for each of the three independent replicates. Errors are represented as the standard deviation derived from the fitting. The sequences for each variant are shown.

Variant	Sequence	k_{cat} (s^{-1})	K_M (mM)	k_{cat}/K_M (s^{-1} M^{-1})
V4 (pH 8.5)	GLRGGG	703 ± 104	6.67 ± 0.65	(1.9 ± 0.04) × 10^5
		606.4 ± 181	3.05 ± 1.14	(2.0 ± 0.1) × 10^5
		596.9 ± 164	2.71 ± 0.85	(2.2 ± 0.2) × 10^5
V4 (pH 7.0)	GLRGGG	299.0 ± 99.8	2.33 ± 1.02	(1.3 ± 0.1) × 10^5
		460.9 ± 53.3	4.54 ± 0.61	(1.02 ± 0.02) × 10^5
		462.6 ± 143.0	3.19 ± 1.22	(1.5 ± 0.1) × 10^5
V3	DIRGGG	206.8 ± 55.3	3.24 ± 1.07	(6.4 ± 0.4) × 10^4
		190.4 ± 54.4	3.18 ± 1.12	(6.0 ± 0.4) × 10^4
		211.4 ± 17.7	3.65 ± 0.37	(5.8 ± 0.1) × 10^4
V6	GLHGGG	93.3 ± 6.5	2.38 ± 0.22	(3.9 ± 0.1) × 10^4
		61.8 ± 7.4	1.31 ± 0.24	(4.7 ± 0.3) × 10^4
		69.1 ± 14.9	1.53 ± 0.48	(4.5 ± 0.4) × 10^4
V1	KLRGGG	53.8 ± 18.2	1.41 ± 0.72	(3.8 ± 0.7) × 10^4
		38.0 ± 2.6	1.01 ± 0.12	(3.8 ± 0.2) × 10^4
		70.4 ± 20.4	1.91 ± 0.76	(3.7 ± 0.4) × 10^4

V2	KSIGGG	49.1 ± 12.8	1.42 ± 0.55	(3.5 ± 0.5) × 10^4
		60.9 ± 5.2	2.18 ± 0.25	(2.8 ± 0.1) × 10^4
		54.1 ± 14.3	1.66 ± 0.63	(3.3 ± 0.4) × 10^4
V5	RGAGGG	55.0 ± 12.5	1.95 ± 0.61	(2.8 ± 0.2) × 10^4
		52.3 ± 15.2	1.86 ± 0.75	(2.8 ± 0.3) × 10^4
		59.0 ± 1.9	2.10 ± 0.09	(2.81 ± 0.03) × 10^4
V8	VGGRFI	61.8 ± 27.0	2.58 ± 1.45	(2.4 ± 0.3) × 10^4
		60.8 ± 34.0	2.47 ± 1.81	(2.4 ± 0.4) × 10^4
		38.0 ± 18.2	1.24 ± 0.95	(3.1 ± 0.9) × 10^4
V7	NNIGGG	41.0 ± 10.6	1.59 ± 0.60	(2.6 ± 0.3) × 10^4
		40.4 ± 11.2	1.39 ± 0.58	(2.9 ± 0.4) × 10^4
		84.8 ± 14.1	4.51 ± 0.88	(1.9 ± 0.1) × 10^4
V9	VGGAPL	20.0 ± 3.0	1.11 ± 0.27	(1.8 ± 0.2) × 10^4
		22.2 ± 3.9	0.94 ± 0.28	(2.4 ± 0.3) × 10^4
		28.9 ± 7.6	1.31 ± 0.53	(2.2 ± 0.3) × 10^4
V10	VGGGTP	17.6 ± 2.4	1.43 ± 0.30	(1.2 ± 0.1) × 10^4
		13.9 ± 2.8	0.74 ± 0.28	(1.9 ± 0.3) × 10^4
		16.1 ± 3.5	1.39 ± 0.45	(1.2 ± 0.1) × 10^4
BACKGROUND	VGGGGG	21.9 ± 4.0	2.35 ± 0.56	(9.4 ± 0.6) × 10^3
		11.3 ± 3.8	1.21 ± 0.65	(9.3 ± 1.9) × 10^3
		15.7 ± 2.3	1.56 ± 0.33	(1.0 ± 0.1) × 10^4

Table S2. Sequences for the mutagenic primers used to saturate three simultaneous positions of the included polypeptide for the generation of the combinatorial libraries.

COMBINATORIAL LIBRARY	PRIMER NAME	PRIMER SEQUENCE
Library 1 (VGGGGG)	VHH_F	gtggtggtggtggtggtgctcgagtgaNNNNNNNNN accacccacccacgccgccacaaccagacgc
	VHH_R	gcgtctggttgtggcggcgtgggtgggtggtNNNNNNNNN tcactcgagcaccaccaccaccaccac
Library 2 (VGGGGG)	HHH_F	gtggtggtggtggtggtgctcgagtgagccaccacc NNNNNNNNNccacgccgccacaaccagacgc
	HHH_R	gcgtctggttgtggcggcgtggNNNNNNNNN ggtggtggctcactcgagcaccaccaccaccaccac
